# Mid-term results of total hip arthroplasty with subtrochanteric Z-osteotomy in Crowe type 3-4 developmental hip dysplasia

**DOI:** 10.3906/sag-2102-217

**Published:** 2021-08-30

**Authors:** Vedat BİÇİCİ, İzzet BİNGÖL, Tamer SAZAK

**Affiliations:** 1 Department of Orthopedics and Traumatology, Ankara City Hospital, Ankara Turkey; 2 Department of Orthopedics and Traumatology, 29 Mayıs State Hospital, Ankara Turkey; 3 Department of Orthopedics and Traumatology, Yıldırım Beyazıt University Yenimahalle Research and Training Hospital, Ankara Turkey

**Keywords:** Developmental dislocation of the hip, subtrochanteric femoral shortening osteotomy, osteotomy fixation, total hip replacement

## Abstract

**Background/aim:**

Total hip arthroplasty (THA) is technically more difficult and has higher complication rates in patients with Crowe type 3–4 developmental dysplasia of the hip (DDH). Due to the difficulties and different treatment options, there is still no consensus on the optimal treatment. We aimed to evaluate the mid-term results of our patients who had undergone subtrochanteric femoral shortening Z-osteotomy.

**Materials and methods:**

This study included 37 hips of 29 patients with the diagnosis of Crowe 3-4 DDH between June 2010 and December 2016 and who underwent femoral shortening Z-osteotomy with cementless total hip arthroplasty. Acetabular component was determined according to the patient’s age and functional capacity and all patients were operated with a posterior approach. Functional results, postoperative complications, Harris and visual analogue scale (VAS) scores were evaluated.

**Results:**

The average Harris hip score was 41.3 ± 3.1 preoperatively and 84.7 ± 4.3 postoperatively (p < 0.05). The mean preoperative hip pain score on the VAS was 7.9 (range: 6–9) and this was significantly lower at the last follow-up (mean: 3.4; range: 2–4) (p < 0.05). The final mean limb-length discrepancy was 1.3 cm. The average amount of femoral shortening was 3.2 cm. Regarding complications, 3 (10.3%) patients had dislocations. These patients underwent closed reduction. Sciatic palsy developed in 1 (3.4%) patient. The patient was reoperated on for sciatic nerve dissection in the early period.

**Conclusion:**

Subtrochanteric shortening Z-osteotomy combined with cementless total hip replacement can be considered an effective and successful method in selected patients with Crowe 3-4 coxarthrosis.

## 1. Introduction

Total hip arthroplasty (THA) is technically more difficult and has higher complication rates in patients with Crowe type 3-4 developmental dysplasia of the hip (DDH) [1]. Unlike primary osteoarthritis, the low bone stock in the original acetabulum and accompanying anatomical abnormalities such as hypoplastic structure, hypertrophied joint capsule, more horizontal and shorter abductor muscles, deformed femoral head, and smaller and narrow femoral structure are the main reasons for this [2]. In addition to all these points, operations performed for osteotomies in childhood (difficulty in placing the femoral component due to obstruction in the femoral canal, alteration of the femoral alignment) can be even more complicated [3]. One of the goals in performing THA is to reduce contact stress and prolong acetabular cup survival by moving the hip’s rotation center to the real acetabulum. It is aimed to equalize the leg length on the femoral side, to place the appropriate femoral component, and to lower the femur without damaging the sciatic nerve [4,5]. However, leg extensions of more than 3–4 cm are associated with an increased risk of sciatica injury. Femoral shortening osteotomy has become a standard approach since it prevents sciatic nerve damage, has lower nonunion rates, protects the proximal femoral metaphysis, and thus provides correction of femoral rotation and enables cementless femoral component placement [6,7]. Transverse osteotomy was first used for this purpose and still continues to be the most commonly used osteotomy technique. However, the centrosymmetry of the intersecting surface increases the rotational instability, which, in combination with smaller surface contact, might lead to nonunion of the osteotomy [4]. Therefore, especially in recent years, focus has been on different techniques such as subtrochanteric Z-osteotomy (step-cut) and double-chevron or oblique osteotomies. Each technique has its own advantages and disadvantages, and different results are reported [8–11]. Although subtrochanteric Z-osteotomy has some advantages such as fewer rotation errors and a larger contact surface for bone union, it is rarely applied compared to other techniques due to lack of experience and the facts that it is technically much more difficult and there are fewer case series in the literature. Due to these difficulties and different treatment options, there is still no consensus on the optimal treatment method for Crowe type 3-4 DDH patients.

In this study, we aimed to evaluate the mid-term results of our patients who had undergone the less frequently applied subtrochanteric femoral shortening Z-osteotomy due to Crowe type 3-4 DDH, thereby making a contribution to the literature.

## 2. Materials and methods

After obtaining the necessary ethics committee approval (approval: E1-20-950) and the informed consent of patients, 37 hips of 29 patients who had been admitted to our clinic with the diagnosis of Crowe type 3-4 DDH between June 2010 and December 2016 and who underwent femoral shortening Z-osteotomy with cementless total hip arthroplasty were enrolled in this study. Their medical files and radiology records were evaluated retrospectively. The study’s inclusion criteria were severe pain unresponsive to conservative treatment, functional impairment and difficulty in walking and daily activities, and Crowe type 3-4 DDH. Exclusion criteria were oncological diseases of the hip, previous hip surgery (trauma, osteotomy), and lack of willingness to participate in the study.

Functional results of the patients were evaluated using preoperative and postoperative Harris hip scores [12]. Hip pain was measured with a visual analogue scale (VAS) [13]. The Trendelenburg sign was used in clinical evaluation for preoperative and postoperative leg length (clinically measured between the spina iliaca anterior superior and the medial malleolus) and muscle strength measurements. In radiological evaluation, anteroposterior and lateral hip radiographs were taken routinely. In addition, polyaxial sections (coronal and sagittal) were evaluated by pelvic computerized tomography (CT) to determine the acetabular structure and bone stock. In all patients, accurate preoperative planning was performed, paying attention to the bone stock and especially the level of subtrochanteric osteotomy. The same surgical procedure was applied for all of our patients. In all cases, the acetabular cup was placed in the true acetabulum. In the radiographic analysis of the prosthesis, the acetabular component was evaluated according to the DeLee and Charnley criteria [14] and the femoral component according to the Gruen criteria [15].

### 2.1. Surgical technique

**Figure 1:**
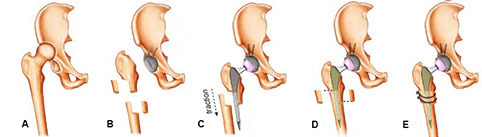
Schematic picture of the steps of the subtrochanteric Z-osteotomy technique.

All of our patients were operated on with a standard procedure by the same surgeon in accordance with our own surgical technique. The patients were placed in the lateral decubitus position with a posterior approach (modified Gibson). The joint capsule was exposed and excised, the femoral head was exposed, and the femoral neck was osteotomized. The soft tissue and osteophytes around the real acetabulum were cleaned and the true acetabulum was exposed. Since there is generally insufficient stock in the medial wall, the acetabulum was prepared with an acetabular reamer without medialization and medial protrusion. The prepared acetabular component was placed in a 1-mm press-fit, and 1–3 acetabular screws of the longest possible size were placed to capture the opposite cortex for better fixation. Before performing femoral osteotomy, the gluteal sling was completely relaxed to prevent possible sciatic nerve injury. Iliopsoas tenotomy was performed in unilateral cases but not in bilateral ones. The osteotomy level was determined from the subtrochanteric region and it was marked by electrocautery to prevent rotation error (Figure 1a). Step-cut femoral osteotomy was then performed with a reciprocating saw (Figure 1b). After the proximal femur was reamed and scraped, the hip was reduced with a trial prosthesis and the amount of shortening according to the overriding of the distal femur was evaluated separately for each patient’s individual case. After femoral shortening (2–4 cm) was performed to the specified amount (the amount of femoral shortening was done by considering the preoperative leg length difference in unilateral patients. It was determined only by intraoperative overriding in patients with bilateral), the distal femur was reamed to the length of the stem, preserving the bone stock as much as possible and ensuring the attachment of the femoral component as tightly as possible. Thus, it was aimed to have the best level of rotational stability and distal involvement (Figure 1c). A cementless femoral stem of the appropriate diameter was placed. For additional rotational stability, the onlay strut femur autograft (Figure 1d), which was removed from the cable, plate screw, and osteotomy line, was divided in half vertically, placed in the fracture line laterally and medially in a semi-tubular form, and fixed with a cable (Figure 1e). The hip was reduced with the acetabular liner and femoral head, and hip stability was checked. In none of our cases was the femoral head used for additional stabilization for the acetabulum (cage, ring, constraint cup) or to support the acetabular roof.

### 2.2. Postoperative care

In the postoperative period, the patients were mobilized with no loads on the next day of the operation. Antirotation boot was used for 3 weeks at night to control involuntary and excessive motions. The patients were followed at the 2nd, 4th, 8th, 12th, and 24th weeks and then invited for annual controls in the postoperative period, being evaluated clinically and radiologically. Routine anteroposterior and lateral hip radiographs were taken. Partial weight-bearing started in the 4th week. Full weight-bearing was started after solid union was observed on the radiographs.

### 2.3. Statistical analysis

SPSS v: 18.0 (SPSS Inc., Chicago, IL, USA) was used for the statistical analysis of the study. The conformity of the data to normal distribution was evaluated by visual (histogram and probability graphs) and analytical (Kolmogorov–Smirnov test) methods. The two-sided, paired Student t-test was used for statistical analysis of the pre- and postoperative Harris and VAS scores. Statistical differences were considered to be significant at p < 0.05.

## 3. Results

**Figure 2:**
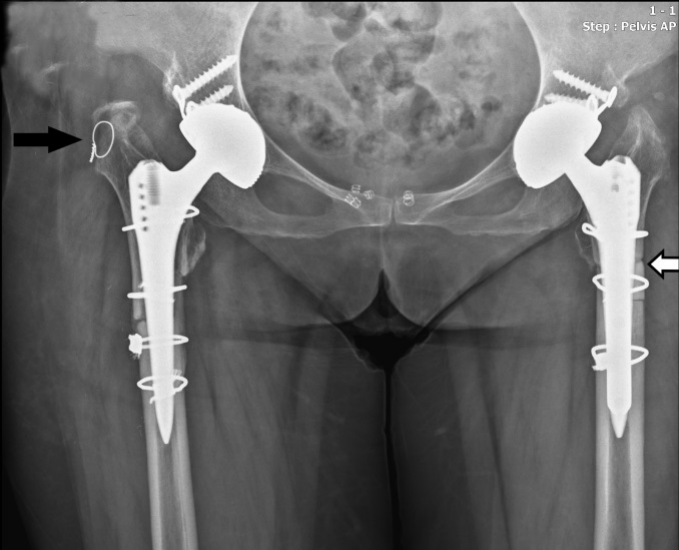
X-ray radiography of patient whose trochanter major fracture (black arrow) was repaired with cerclage with a fracture that developed in the intraoperative osteotomy line (white arrow).

**Figure 3:**
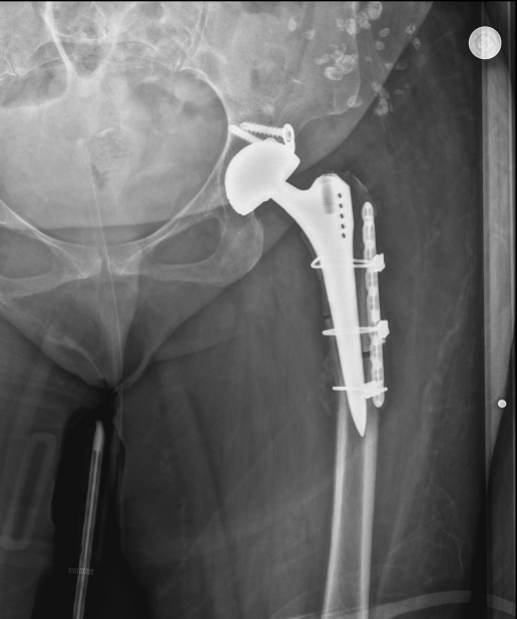
Early postoperative X-ray radiograph of patient who underwent osteotomy fixation with plate and cable.

**Figure 4:**
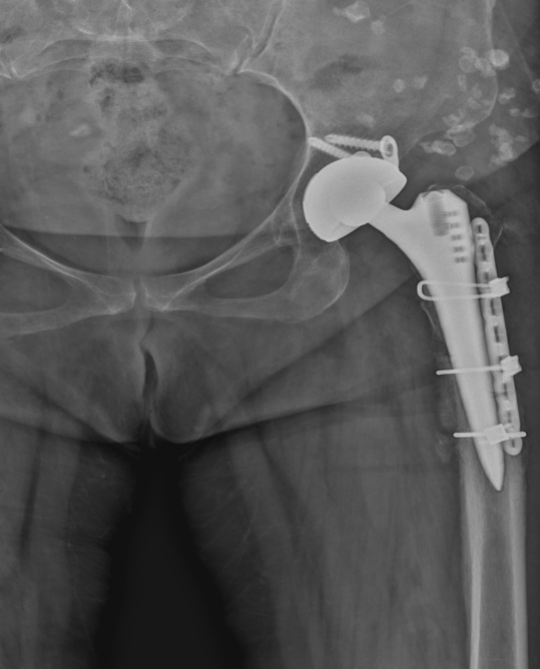
X-ray radiograph of patient who underwent osteotomy fixation with plate and cable after union.

The average age of our patients was 43 ± 9.8 (30–58) years; 21 of the patients were women and 8 were men. While 3 patients were Crowe type 3, 26 patients were Crowe type 4. Our longest follow-up period was 11 years, the shortest was 6 years, and the average was 8.3 years. Eight (27.5%) of our patients were treated bilaterally. The average Harris hip score was 41.3 ± 3.1 preoperatively and 84.7 ± 4.3 postoperatively (p < 0.05). The mean preoperative VAS score was 7.9 (range: 6–9), and this was significantly lower at the last follow-up (mean: 3.4; range: 2–4) (p < 0.05). The mean preoperative limb-length discrepancy in unilateral cases was 5.7 cm (range: 3–8 cm). After surgery, the final mean limb-length discrepancy was 1.3 cm (range: 0–2.6 cm). The average amount of femoral shortening performed for patients was 3.2 cm. The mean operation time was 72 ± 14.3 min (range: 55–94). Preoperatively, the Trendelenburg sign was present in all patients, while at postoperative final controls, it was detected in 3 patients (10.3%). In one patient intraoperatively, a fracture occurred in the left femur osteotomy line, but it was fixed with a cable. The same patient had an avulsion fracture in the right trochanter major. It was repaired with cerclage wire. It was found that there was improvement and no problems in the follow-up (Figure 2). As an early complication, 3 (10.3%) patients had dislocations. These patients underwent closed reduction. No re-dislocation was observed after reduction. Sciatic palsy developed in 1 (3.4%) patient. The patient was reoperated on for sciatic nerve dissection in the early period. Intraoperative evaluation revealed insufficient loosening of the gluteal sling. In the follow-up of that patient, the sciatic nerve area had partially recovered. All of our patients had union in the osteotomy line and femoral stem revision was not performed. For the fixation of the osteotomy line, only cable was used in 22 hips, while onlay graft and cable were used in 14 hips. In only 1 hip, the fracture line was fixed with plate and cable because the osteotomy line was too distal and the femoral stem was relatively short (Figure 3). In the follow-up of that patient, it was found that the osteotomy line had healed with a slight varus deformity in the femur (Figure 4). Protrusion of the acetabular cup developed in 1 (3.4%) patient and it was revised with an acetabular cage. Our average prosthesis survival rate was 97.2%.

### 3.1. Components and fixation

Different types of prostheses were used for both the cup and the stem. The median size of the acetabular component for the EP-FIT PLUS (Smith & Nephew Orthopaedics AG, Baar, Switzerland) was 42 mm (range: 40–46 mm), while for the POLARCUP Dual Mobility System (Smith & Nephew Orthopaedics AG), it was 43 mm (range: 43–47 mm). The most widely used stems were SL-PLUS™ stems (Smith & Nephew Orthopaedics AG). The other applied stems were Synergy stems (Smith and Nephew, Memphis, TN, USA) (Table).

**Table:** Implant types and sizes used.

	Number (%)	Sizes of implants
Femoral stemsSL-PLUS Synergy	30 (81%)7 (19%)	Size 01: 21 pieces, Size 0: 7 pieces, Size 1: 2 piecesSize 9: 5 pieces, Size 10: 2 pieces
Acetabular componentsEP-FIT PLUSPOLARCUP	9 (24.3%)28 (75.7%)	40 mm: 3 pieces, 42 mm: 4 pieces, 44 mm: 1 piece, 46 mm: 1 piece43 mm: 19 pieces, 45 mm: 7 pieces, 47 mm: 2 pieces

## 4. Discussion

Femoral shortening THA is one of the most challenging hip surgeries because of its more difficult surgical technique and higher complication rates compared to primary THA [16]. Therefore, preoperative planning should be done carefully and all possible risks should be evaluated. In order to achieve good postoperative results, the most appropriate surgical treatment should be applied to normal anatomical points. The first important problem to be tackled in cases of Crowe type 3–4 DDH is the optimal placement of the acetabular cup for the restoration of the rotation center. Although some studies reported good results with the placement of the cup in the new acetabulum [17,18], the best outcomes in terms of the length of the leg, stability, and biomechanical results are obtained by placing the cup in the original acetabulum [19,20]. The most striking result of our study was that the cup was placed in the real acetabulum for all of our patients and the hip rotation center was placed in its natural location. Thus, biomechanical and functional anatomical restoration was provided. In addition, placing the cup in the real acetabulum also has an effect on prosthesis survival. Nagoya et al. did not detect acetabular loosening in any of their cases during a mean follow-up period of 8.1 years [21]. Similarly, Rollo et al. reported 100% acetabular survival in a mean follow-up of 88 months (range: 63–133) [22]. In our study, acetabular loosening was not observed in any of our patients during our follow-up period, and results were consistent with the literature.

One of the other common problems encountered during operations in cases of Crowe type 3-4 DDH is the lack of sufficient bone stock in the acetabulum when placing the acetabular component; the desired coverage cannot be achieved due to its shallow structure. To solve this problem, different methods such as fixation of autologous femoral head grafts or support with allogeneic bulk grafts are applied for the acetabular roof. All of these methods are aimed at increasing the acetabular bone stock. Although there are publications in the literature reporting successful results, good graft cooperation, and normal revision rates [23,24], Mulroy et al. and Rodriguez et al. stated that serious problems such as graft lysis, migration and loosening of the acetabular component, and insufficiency of the additional implants used for fixation may develop [25,26]. In none of the cases in our study was the acetabular cup fixed with 2 or 3 screws without using auto-allografts or similar products to support the acetabular structure, and it was aimed to reduce possible complications.

**Figure 5:**
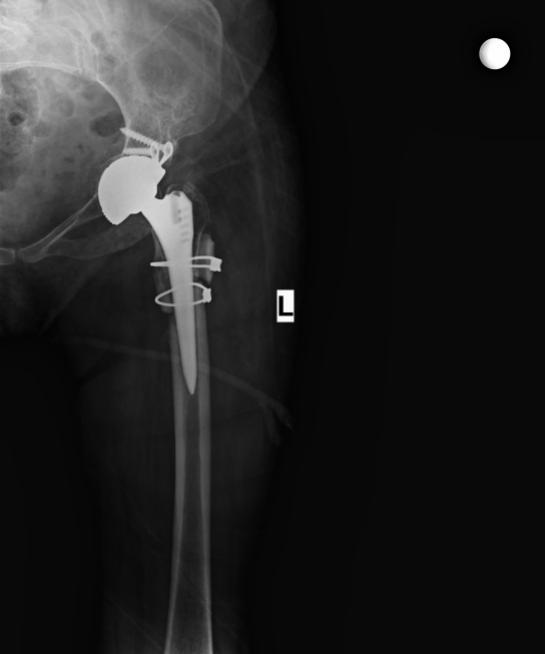
Postoperative X-ray radiograph of patient who underwent osteotomy fixation with cable and onlay strut graft.

**Figure 6:**
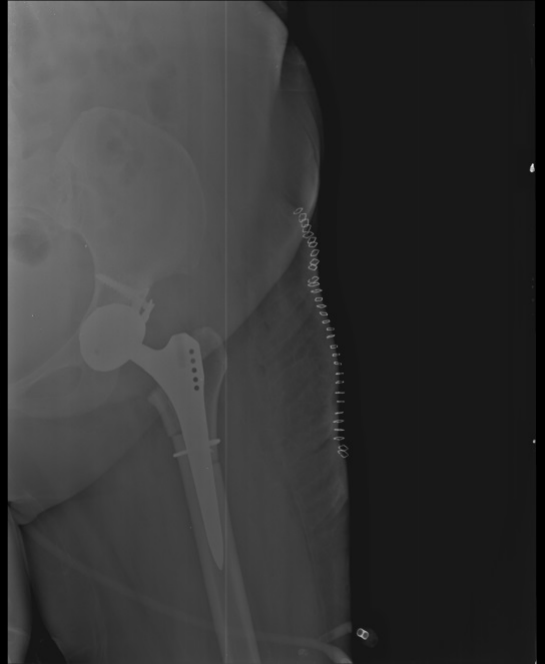
Postoperative X-ray radiograph of patient who underwent osteotomy fixation with only a cable.

Many different results have been reported regarding the level of femoral shortening osteotomy (proximal, shaft, and distal). Proximal femoral osteotomies have become more popular, especially due to additional morbidities such as nonunion, the need for additional incision, and, therefore, the formation of a larger wound area and prolonged operation time [27]. In proximal osteotomies, different techniques such as transverse and modified osteotomies (oblique, double chevron, z-shape) have been defined in general. These techniques have been used to increase the contact surface to facilitate union in the osteotomy line, to provide rotational stability, and to provide better load transfer. The advantages and disadvantages of each osteotomy technique have been reported. Transverse osteotomy has been used most frequently in the literature due to its ease of application and shortening of the surgical time, and successful results have been reported [28,29]. However, similar results have been reported for other modified osteotomies [30,31]. Because of these similar results, the most ideal osteotomy is not clear. Li et al. [32] compared transverse and modified osteotomies in the most comprehensive meta-analysis in the literature on proximal osteotomies. The results showed that method of osteotomy was not associated with nonunion rate, as well as other post-operative outcomes including nerve palsy, dislocation, revision, leg-length discrepancy, HHS improvement, and deep infection. Modified osteotomies appear to be more advantageous in terms of rotational stability. They reported that an objective evaluation could not be made due to different features such as the design differences of the femoral stems used in the studies, the coatings made in different sizes and techniques. In addition, in proximal osteotomies, the fixation of the osteotomy line is also a difficult and problematic process, since the femoral stem is located in the femur medulla. Stable fixation for the healing of the osteotomy line, early weight-bearing, and rotational stability are among the main goals. Plate and screw fixation with the femoral stem itself, cable fixation only, and cable fixation with autogenous grafts are among the methods applied without using additional implants [33]. It is aimed to use the least possible additional implant, as it extends the duration of the operation and increases the negative effects and costs of recovery. Bao et al. [34] reported that they achieved sufficient fixation without using additional implants by applying a cylindrical prosthesis with proximal attachment and distal cleft. Çatma et al. [35], in their study comparing cases in which plate-screw and autogenous graft-cable fixation was used, found earlier union in the osteotomy line and significant decrease in the duration of surgery in patients who underwent autogenous graft-cable fixation. Muratli et al. [36] found that intramedullary stability was primarily related to the cross-sectional geometry of the femoral stem in their cadaver study, where they compared 4 different osteotomies: biomechanically transverse, oblique, Z-subtrochanteric, and double-chevron. It was determined that rotational stability increased even more with allogeneic strut grafts and cables. They concluded that Z-shape osteotomy is more successful in antetorsion, rotation, and angulation and that it should be used for patients with good bone stock and body mass index of less than 28. For the remaining cases, they stated that transverse osteotomy should be the second option. Autogenous graft-cable was used for fixation of the osteotomy line in all our cases except one, where the osteotomy line, for which we had to use plates and cables, was made very distal and the femoral stem was short (Figure 5), or we applied fixation only with a cable (Figure 6). Since we did not use additional fixation methods in the acetabulum and fixed the osteotomy line in the femur with autograft and cable, we tried to keep the operation time shorter and the operation cost was also lower.

Our study has several limitations. The first and most important limitation is the lack of a control group in our study. Second, we have reported mid-term results. Long-term results would provide more reliable information. Third, our study lacked measurements of muscle forces and evaluations of walking mechanisms. That would give more valuable information about walking mechanisms and hip functions.

In conclusion, subtrochanteric shortening Z-osteotomy combined with cementless total hip replacement can be considered an effective and successful method in selected patients with Crowe type 3–4 coxarthrosis. This procedure allows the restoration of a more normal limb length with good clinical outcomes, a low rate of neurological and other complications, and high prosthesis survival rates.
